# Mechanisms of growth inhibition of primary prostate epithelial cells following gamma irradiation or photodynamic therapy include senescence, necrosis, and autophagy, but not apoptosis

**DOI:** 10.1002/cam4.553

**Published:** 2015-11-21

**Authors:** Fiona M. Frame, Huguette Savoie, Francesca Bryden, Francesca Giuntini, Vincent M. Mann, Matthew S. Simms, Ross W. Boyle, Norman J. Maitland

**Affiliations:** ^1^ YCR Cancer Research UnitDepartment of BiologyUniversity of YorkHeslingtonNorth YorkshireYO10 5DDUnited Kingdom; ^2^ Department of ChemistryUniversity of HullKingston Upon HullHU6 7RXUnited Kingdom; ^3^ School of Pharmacy and Biomolecular SciencesLiverpool John Moores UniversityLiverpoolL3 2AJUnited Kingdom; ^4^ Department of UrologyCastle Hill Hospital (Hull and East Yorkshire Hospitals NHS Trust)CottinghamHU16 5JQUnited Kingdom; ^5^ Hull York Medical SchoolUniversity of HullHullHU6 7RXUnited Kingdom

**Keywords:** Cancer stem‐like cells, photodynamic therapy, prostate cancer, radiotherapy

## Abstract

In comparison to more differentiated cells, prostate cancer stem‐like cells are radioresistant, which could explain radio‐recurrent prostate cancer. Improvement of radiotherapeutic efficacy may therefore require combination therapy. We have investigated the consequences of treating primary prostate epithelial cells with gamma irradiation and photodynamic therapy (PDT), both of which act through production of reactive oxygen species (ROS). Primary prostate epithelial cells were cultured from patient samples of benign prostatic hyperplasia and prostate cancer prior to treatment with PDT or gamma irradiation. Cell viability was measured using MTT and alamar blue assay, and cell recovery by colony‐forming assays. Immunofluorescence of gamma‐H2AX foci was used to quantify DNA damage, and autophagy and apoptosis were assessed using Western blots. Necrosis and senescence were measured by propidium iodide staining and beta‐galactosidase staining, respectively. Both PDT and gamma irradiation reduced the colony‐forming ability of primary prostate epithelial cells. PDT reduced the viability of all types of cells in the cultures, including stem‐like cells and more differentiated cells. PDT induced necrosis and autophagy, whereas gamma irradiation induced senescence, but neither treatment induced apoptosis. PDT and gamma irradiation therefore inhibit cell growth by different mechanisms. We suggest these treatments would be suitable for use in combination as sequential treatments against prostate cancer.

## Introduction

When watchful waiting or active monitoring is not an option, the most common therapies for localized prostate cancer include brachytherapy, radiotherapy, and surgery to remove the entire prostate gland (radical prostatectomy) 1, 2, 3, 4. All these have side effects, including potential damage to other organs and healthy tissue, incontinence, and impotence. Unfortunately, in the case of prostate cancer, men are being overtreated due to our inability to distinguish between low‐risk disease and aggressive cancers 5. As such, there is a need for precise focal therapies including cryotherapy, high intensity focused ultrasound (HIFU), and photodynamic therapy (PDT) 6. PDT is currently in use for several tumor types, including head and neck cancer 7, 8, lung cancer 9, skin cancer, 10, 11 and more recently, prostate cancer 12. PDT is emerging as one of a new series of treatment options that aim to focally ablate prostate cancer, thus eliminating the tumor(s) with fewer side effects 12, 13. Since improved screening methods should lead to earlier detection of prostate tumors, improved imaging methods will allow focal therapy to become increasingly targeted 14. In addition, focal therapies may now be applicable in combination with more standard therapies, such as chemotherapeutic agents and radiotherapy 15.

Although PDT is already being used to treat prostate cancer in the clinic, the biological basis for its effectiveness has not been fully explored, and although there are some data on prostate cell lines 16, 17, 18, the effect of PDT on primary cells cultured from patient samples has not been reported. In this study, we used a series of low passage primary prostate epithelial cells cultured directly from patient tissue, and treated them with a porphyrin‐based photosensitizer, which can be conjugated to targeting peptides and proteins 19, 20, 21. However, in this in vitro study we were simply interested in determining the potential of the core photosensitizer to eliminate prostate cancer cells upon light irradiation, so the chemical group previously used for conjugation was prereacted with a simple amine to prevent any nonspecific conjugation occurring during the experiments. Significantly, we also tested the effect of this photosensitizer drug on the prostate stem‐like cell (benign and malignant) population that are already known to be radio‐resistant 22 and are proposed to give rise to secondary tumors. Alongside the PDT photosensitizer, we also tested the effect of gamma irradiation on primary prostate epithelial cells. Radiotherapy is a widely used treatment for prostate cancer, however, 30% of patients treated in this way will experience disease recurrence 23. Whilst we have determined one mechanism of radio‐resistance in primary prostate epithelial cells, increased heterochromatin in the stem‐like cell population, there may be multiple mechanisms. Although both therapies produce reactive species, our aim was to determine the mechanism of action of each therapy.

## Materials and Methods

### Culture of primary prostate epithelial cells from patient tissue

Benign prostatic hyperplasia (BPH) and prostate cancer samples were obtained from TURP (transurethral resection of the prostate) and radical prostatectomy operations. Prostate epithelial cells were cultured as described 24 in stem cell media (SCM). Table 1 shows the samples used, including patient and pathology information.

**Table 1:** Patient samples

Sample	Passage	Operation	Patient age	Diagnosis	Hormone status
Benign samples					
H109/11	p3‐p4	T	77	Benign	
H076/11	p3‐p4	T	63	Benign	
H398/14	p4	T	66	Benign	
Y053/11	p4‐p5	T	78	Benign	
Y068/09	p3	T	–	Benign	
H059/11	p3‐p5	T	74	Benign	
H340/13 Lb	p4‐p5	R	59	Normal	
Cancer samples					
H253/12 La	p4	R	71	Cancer Gl7 (4 + 3)	Naive
H263/12 Lb	p3	R	58	Cancer Gl7 (3 + 4)	Naive
569	p5	R	67	Cancer Gl8 (3 + 5)	Naive
H316/13 La	p3‐p6	R	61	Cancer Gl7 (3 + 4)	Naive
H317/13 La	p4‐p5	R	65	Cancer Gl7 (4 + 3)	Naive
H290/13 Ra	p3‐p4	R	69	Cancer Gl7 (4 + 3)	Naive

R, radical prostatectomy; T, trans‐urethral resection of the prostate.

### Ethics statement

Patient samples were collected with ethical permission from York District Hospital (York) and Castle Hill Hospital (Cottingham, Hull) (Ethics Number: 07/H1304/121). Use of patient tissue was approved by the Local Research Ethics Committees. Patients gave informed consent and all patient samples were anonymized.

### Selection of cells

Cells were trypsinized, resuspended in SCM and plated on collagen I‐coated plates following blocking by 0.3% heat‐inactivated BSA. After 20 min, cells that remained unbound were harvested, together with cells from three PBS washes; this constituted the committed basal cells (CBs) that do not rapidly adhere to collagen I. The bound cells were trypsinized, resuspended in MACs buffer (PBS/2 μmol/L EDTA/0.5% FCS) and incubated with CD133‐microbeads. The CD133 microbead kit and MACs columns (130‐050‐801, 130‐042‐201, Miltenyi Biotec, Surrey, UK) were used to select the CD133^+^ and CD133^−^ cells. The three cell populations obtained were as follows: stem‐like cells (SC)—*α*2*β*1integrin^hi^/CD133^+^, transit‐amplifying cells (TA)—*α*2*β*1integrin^hi^/CD133^−^, and committed basal cells (CB)—*α*2*β*1integrin^lo^. Significantly, the SC population is very rare and following selections from millions of cells from the total population the yield is a few hundred to a few thousand SCs (~0.01%).

### Preparation of photosensitizer

5‐[Aminobutyl‐N‐oxycarbonyl)phenyl]phenyl]‐10,15,20‐tris(N‐methyl‐4‐pyridinium)porphyrin trichloride was prepared as follows:

To a solution of 5‐[4‐(succinimide‐N‐oxycarbonyl)phenyl]‐10,15,20‐tris‐(4‐N‐methylpyridimiumyl)porphyrin trichloride 20 (50 mg, 0.055 mmol) in dry DMSO (5 mL), butylamine (50 mg, 0.68 mmol) was added and the mixture stirred at room temperature overnight. Water (20 mL) and ammonium hexafluorophosphate were added to the mixture until precipitation of the porphyrin was observed. The precipitate was collected by filtration and redissolved in acetone. Tetrabutylammonium chloride was added to the mixture until precipitation of the porphyrin was observed, and the precipitate collected by filtration. The crude product was purified by precipitation from diethyl ether over methanol to yield the product as a dark purple solid (34 mg, 71.4%).

UV–vis (H_2_O): *λ*max, nm 422, 521, 560, 580, 645. Log *ε* (422 nm) = 5.46. ^1^H‐NMR (DMSO‐d_6_): *δ* 1.01 (t, 3H, J = 8.00 Hz, CH_3_‐CH_2_), 1.43–1.50 (m, 2H, CH_2_), 1.54–1.60, (m, 2H, CH_2_), 1.63–1.71 (m, 2H, CH_2_), 4.72–4.77 (m, 9H, N‐CH_3_), 8.30–8.40 (m, 4H, 5‐o,m‐Ph), 8.94–9.23 (m, 14H, *β*H and 10,15,20‐o‐Py), 9.49–9.57 (m, 6H, 10,15,20‐m‐Py). ^13^C‐NMR (DMSO‐d_6_): *δ* 14.46, (CH_3_‐CH_2_), 20.35, 31.97, 48.37 (N‐CH_3_), 115.31, 116.03, 122.54, 126.63, 132.73 (*β*‐C), 134.73, 135.14, 143.46, 144.78 (*β*‐C), 157.02, 166.43 (C=O). MS: (ESI) m/z 380 (100[M ‐ 3Cl]2+), HRMS: calcd. for C_49_H_44_N_8_O_1_: 380.1814 found 380.1815.

### Gamma irradiation

To irradiate cells, an RS2000 X‐Ray Biological Irradiator containing a Comet MXR‐165 X‐Ray Source (Rad‐Source Technologies Inc., Suwanee, GA) was used. A dose of 2, 5, 10, 25, 50 or 75 Gy was administered.

### Treatment of cells with photosensitizer

Concentrations of PDT drug between 50–5 *μ*mol/L (Conc 1–50 *μ*mol/L, Conc 2–37.5 *μ*mol/L, Conc 3–25.0 *μ*mol/L, Conc 4–12.5 *μ*mol/L Conc 5–8.75 *μ*mol/L, Conc 6–5 *μ*mol/L) were used for the MTT assays. Briefly, 800 *μ*L of the cells (between 4 × 10^5^ and 1 × 10^6^/mL) was added to 200 *μ*L of six dilutions of the photosensitizer in 12 × 75 mm sterile tubes. The tubes (with tops partially open to allow gas exchange) were incubated for 1 h at 37°C and 5% CO_2_, after which the cells were washed with excess medium to eliminate any unbound photosensitizer. The pellets of cells and porphyrin were resuspended in 1 mL medium and 4 × 100 *μ*L of each concentration was dispensed into two 96‐well plates. One plate was irradiated to a dose of 18 J/cm^2^ using a Paterson Lamp BL1000A (Photo Therapeutics Ltd, London, UK—no longer in production) equipped with a red filter (GLEN S100 367 0134: flat response between ~620 and 642 nm). The irradiation dose was determined using a Macam Portable Radiometer model R203, Macam Photometrics Ltd., Livingston, Scotland, UK. The second plate served as a dark control. After light irradiation, the plates were returned to the incubator overnight. After 18–24 h, an MTT cell viability assay was performed and the results expressed as % cell viability versus porphyrin concentration; an IC50 was determined from the resulting curves. Due to a limitation of primary cell cultures (finite number of passages), experiments were primarily done as biological replicates rather than technical replicates.

### MTT assay

Cell viability was determined using an MTT (3‐[4, 5‐dimethylthiazol‐2‐yl]‐2,5 dipheyltetrazolium bromide) colorimetric assay. Briefly, 10 *μ*L of 12 mmol/L MTT solution was added to each well and incubated for 1–4 h at 37°C to allow MTT metabolism. The crystals were dissolved by adding 150 *μ*L of acid‐alcohol mixture (0.04 mol/L HCl in absolute 2‐propanol). The absorbance at 570 nmol/L was measured on a Biotek ELX800 Universal Microplate Reader, Corgenix Ltd, Peterborough, UK and the results expressed relative to control values.

### Alamar blue assay

Rezasurin sodium salt (Sigma–Aldrich, Cambridge, UK—R7017) was used to carry out alamar blue assays. A 25 mmol/L stock was diluted 50‐fold to generate a 10× working stock. Cells were plated at the stated number (1 × 10^4^–2 × 10^4^) per well for whole populations and 100–300 per well for selected subpopulations) in 96‐well plates and incubated with drug (one plate light‐irradiated and a replica plate as dark control). After 24 h, the alamar blue assay to determine cell viability was carried out. One‐tenth volume of the 10× working stock (20 *μ*L in 200 *μ*L) was added to cells in a 96‐well plate and incubated for 2 h. Fluorescence was measured using a BMG Labtech POLARstar OPTIMA microplate reader, BMG Labtech, Ortenberg, Germany.

### Clonogenic assay

To determine long‐term cell recovery following drug treatment, cells were treated as described above (incubated with drug then excess drug washed out), or irradiated with gamma irradiation at the stated doses, and cells plated at 200–500 cells per well in a 24‐well plate (in triplicate), with STO feeder cells. Cells were fed every 2 days and fixed and stained with crystal violet at 7 days. Colonies were counted and plotted as % surviving fraction with untreated cells being set at 100% viable.

### ROS‐Glo H_2_O_2_ assay

In order to measure production of H_2_O_2_, one of the most stable reactive oxygen species and the end product of other ROS enzyme reactions 25, the ROS‐Glo H_2_O_2_ Assay (Promega G8820, Southampton, UK) was carried out. Cells were plated in a 96‐well plate (4000–8000 cells per well for whole population and 100–300 cells per well for selected subpopulations). Drug was incubated with the cells for 1 h. Media were changed and one plate irradiated (with one as a dark control). Following irradiation, H_2_O_2_ substrate from the assay was added and cells incubated for 3 h. 50 *μ*L of media was transferred to a white plate and combined with the luciferin agent. Luminescence was then read in a BMG Labtech POLARstar OPTIMA microplate reader. The original plate could subsequently also be used in an alamar blue assay at 24 h. These assays were multiplexed when using selected cell populations, due to small cell numbers.

### Immunofluorescence

Following treatment, cells were seeded onto 8‐well collagen I‐coated chamber slides for DNA damage detection by *γ*H2AX staining. Cells were washed with PBS and fixed and stained as described previously 22. Antibodies used were antiphospho‐histone H2A.X (Ser139), clone JBW301, Millipore, Cat no. 05– 636, 1:1000 and goat anti‐mouse Alexa Fluor 568, Invitrogen, Paisley, Scotland, UK, Cat no. A‐11004, 1:1000. Slides were mounted using Vectashield with DAPI (Vector Laboratories, Peterborough, UK, Cat no. H‐1200). Images were captured using a Nikon Eclipse TE300 fluorescent microscope (Nikon, Surrey, UK) with a ×63 oil immersion lens.

### Necrosis assay

Cells were incubated with PDT drug for 1 h, then the drug‐containing media were removed and replaced with new media. The cultures were light‐irradiated (with control dark plates), washed with PBS, then incubated with staining solution (Hoechst 33342 (5 *μ*g/mL), Propidium Iodide (PI) (2 *μ*g/mL) and RNAse (100 *μ*g/mL) in PBS) 30 min post light irradiation, for 15 min in the dark at 37°C. Hoechst‐stained blue cells (total cell count) and PI‐stained red cells (necrotic cells) were visualized with a Nikon Eclipse TE300 fluorescent microscope (Nikon, Surrey, UK) using a ×10 lens and quantified using Volocity software (PerkinElmer, Coventry, West Midlands, UK). More than 100 cells per treatment were counted. Data were plotted as the percentage of PI‐positive cells.

### Senescence assay

Primary prostate epithelial cell cultures were irradiated with single or multiple doses, as indicated in the Figure, and incubated for 48 h before staining for *β*‐galactosidase (pH 6.0) to measure senescence. The Senescence *β*‐Galactosidase Staining Kit was used according to manufacturer's instructions (Cell Signaling Technology, Hitchin, Herts, UK Cat no. 9860).

### Western blotting

Following treatment and light irradiation with the PDT drug or gamma irradiation, cell lysates were harvested using Cytobuster Protein Extraction Reagent (71009, EMD Millipore, Darmstadt, Germany) with protease inhibitors (cOmplete, EDTA‐free Protease Inhibitor Cocktail Tablets, Roche Applied Science, West Sussex, UK). 20 *μ*g extracts of cell protein were loaded on 15% SDS‐PAGE gels and wet‐transferred to a PVDF membrane. Antibodies used include: rabbit monoclonal antiactin 1:5000 (04‐1040, millipore), anti‐LC3B 1:3000 (Ab51520, abcam, Cambridge, UK), cleaved caspase‐3 (Asp175) 1:1000 (9661S, Cell Signaling Technology). Secondary antibodies used were anti‐mouse and anti‐rabbit IgG HRP‐linked 1:5000 (Cell Signaling Technology Inc. 7076P2 and 7074S). Kaleidoscope protein marker was run on each gel (161‐0324, Bio‐Rad). 1 *μ*mol/L of staurosporine (24 h incubation) was used as a positive control.

### Statistical analysis

**Figure 1:**
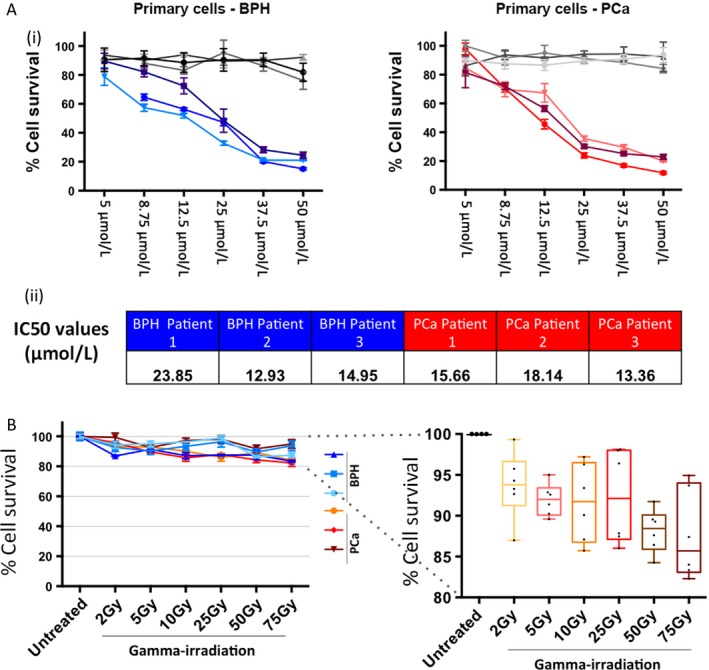
Primary prostate epithelial cells are sensitive to treatment with photodynamic therapy (PDT) drug, but not gamma irradiation when cell survival is measured using a cell metabolism assay. (A) Primary prostate epithelial cells from six patients (3× Benign prostatic hyperplasia (BPH) and 3× PCa) were treated with several doses of PDT drug and light‐irradiated (colored lines), or not light‐irradiated (dark control) (gray lines) and measured using MTT assay after 24 h (i). IC50 values of all patient samples were calculated (ii). (B) Primary prostate epithelial cells from six patients (3 × BPH and 3 × PCa) were treated with several different doses of gamma irradiation and measured using alamar blue assay after 24 h.

MTS and alamar blue assays were performed in triplicate and data presented as the % cell viability with percentage standard error. IC_50_ values (Fig. 1B) were calculated from graphs of transformed data following application of the nonlinear regression (curve fit) that represents the log(inhibitor) ‘v’ normalized response (GraphPad Prism 6 software, La Jolla, CA, USA). For significance calculations, the unpaired, nonparametric Mann–Whitney *U*‐test was used. The *P*‐values indicating statistical significance are displayed (*P < 0.05, **P < 0.01, ***P < 0.001).

## Results

### Cell viability of primary prostate epithelial cells is reduced by PDT drug treatment but not gamma irradiation

Primary epithelial cells cultured from three BPH and three prostate cancer (Gleason 7) patient samples were treated with a range of concentrations of photosensitizer drug (PDT drug), and assessed by MTT assay at 24 h post treatment (Fig. 1A). Nonlight‐irradiated plates were used as a dark control (drug only/no exposure to light). The PDT drug with light irradiation was very effective at reducing viability, with a 75–85% reduction in BPH cells and an 80–90% reduction in cancer cells at the highest concentration. IC_50_ values were calculated for each patient sample and these ranged between 13–24 *μ*mol/L in BPH samples and 13–18 *μ*mol/L in cancer samples (Fig. 1A). Cell viability assays were also carried out following treatment with a range of gamma irradiation doses (0 Gy – 75 Gy) (Fig. 1B). There was very little reduction in viability assay with, at most, a 10–20% reduction. Although initial viability experiments with the photosensitizer used the MTT assay, this was changed to alamar blue because alamar blue is more sensitive for small cell numbers, which was required for later experiments with selected cell populations. The MTT and alamar blue assays are comparable 26, and so it was not felt necessary to repeat the initial MTT assays using alamar blue.

### Both PDT drug and gamma irradiation treatment reduces colony‐forming potential of primary prostate epithelial cells, which correlates with ROS production

**Figure 2:**
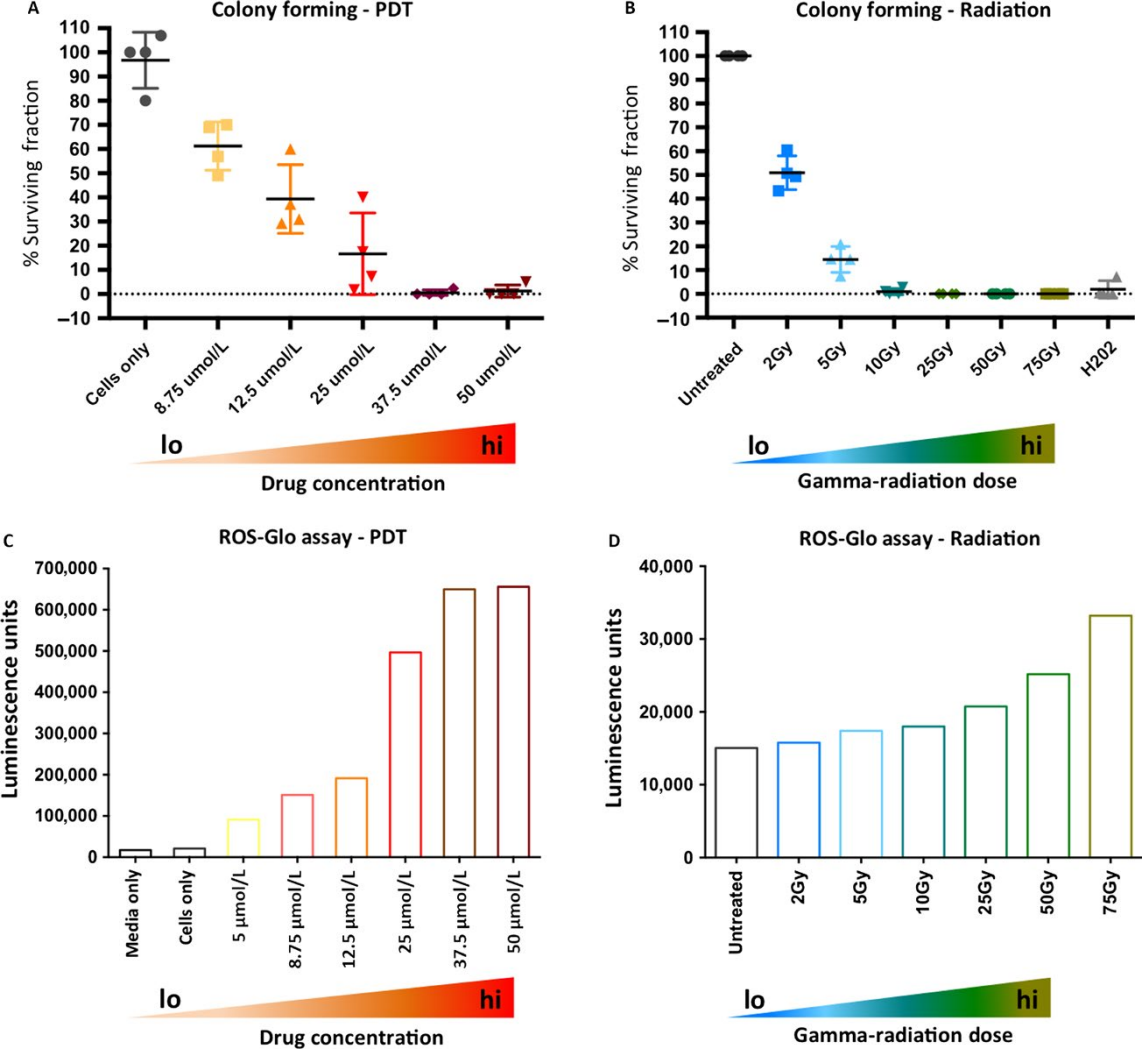
Increasing doses of photodynamic therapy (PDT) drug or gamma irradiation results in a reduction and ablation of colony‐forming ability in primary prostate epithelial cells and an increase in production of Reactive Oxygen Species (ROS). Primary prostate epithelial cells from four patients were treated with varying doses of PDT drug (A) or gamma irradiation (B), and the colony‐forming ability was assessed after 7 days of growth. Colony‐forming ability is presented as % surviving fraction. Primary prostate epithelial cells were treated with increasing doses of (C) PDT drug or (D) gamma irradiation and the production of ROS was measured using the ROS‐Glo assay (Promega).

Colony‐forming assays were carried out following treatment with both PDT drug and gamma‐radiation treatment (Fig. 2), on four samples per treatment type. In both cases, there was a dose‐dependent decrease in colony‐forming ability. With the PDT drug plus light irradiation, concentrations of 37.5 *μ*mol/L and above resulted in complete absence of colonies (Fig. 2A). For the gamma irradiated cells, 10 Gy and higher completely destroyed colony‐forming ability (Fig. 2B). Individual sample graphs are shown in Figure S1, where there is some variation between patients. Therefore, even though there was no dramatic cytotoxicity, as measured by the cell viability assay following gamma irradiation (Fig. 1C), the cells that remained viable had nevertheless lost their colony‐forming ability.

Since both gamma irradiation and photodynamic therapies produce reactive oxygen species (ROS) we measured H_2_O_2_ production in both cases (Fig. 2). PDT showed a dose‐dependent increase in ROS with a maximum of 600,000 luminescence units (Fig. 2C). Gamma irradiation also induced ROS, but with a maximum of 35,000 luminescence units (Fig. 2D). Taking into account the number of cells used in each assay, PDT drug treatment induced 10‐fold more ROS than gamma irradiation.

### PDT drug induces ROS and decreases viability of all selected cell subpopulations in primary prostate epithelial cell cultures

**Figure 3:**
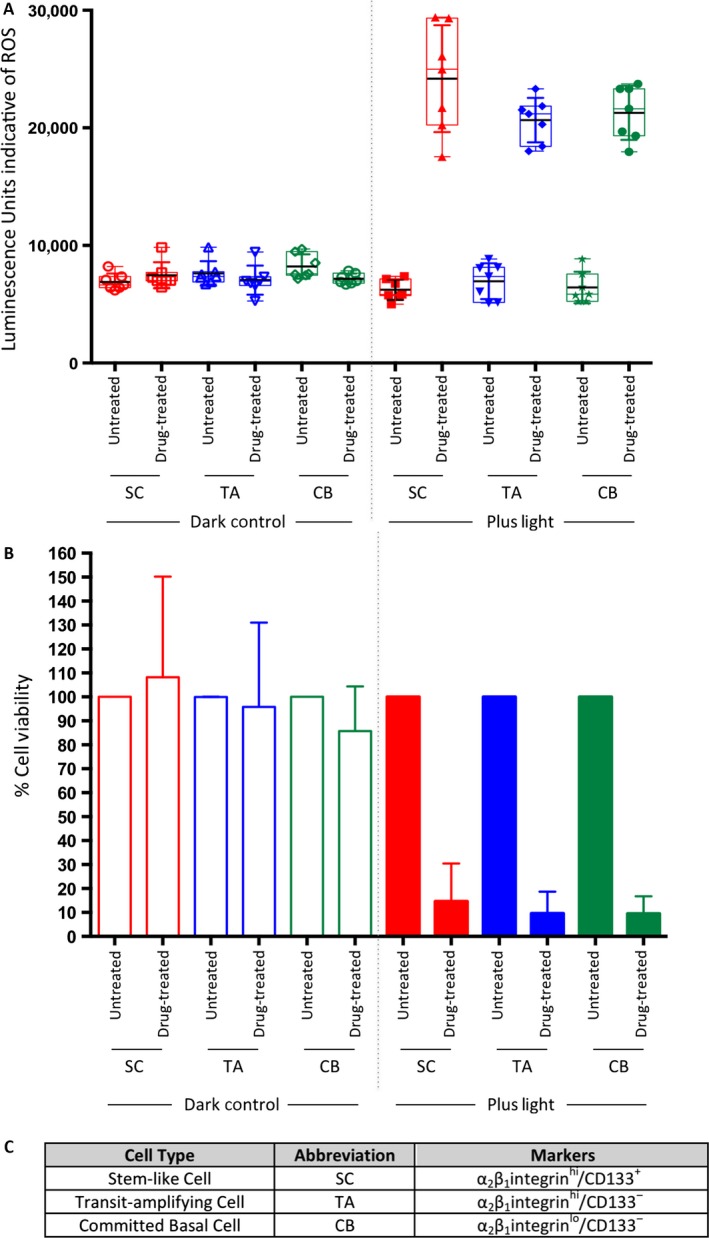
Treatment of selected populations of primary prostate epithelial cells with one dose of photodynamic therapy (PDT) drug results in an increase in reactive oxygen species (ROS) production and decrease in cell viability in all populations. Selected populations of primary prostate epithelial cells were treated with PDT drug (12.5 *μ*mol/L) and (A) ROS production was measured as well as (B) cell viability. (C) List of cell subpopulations and markers.

Previously we had shown that stem‐like cells in primary prostate epithelial cultures showed gamma irradiation resistance when compared with more differentiated transit amplifying and committed basal cells 22. We therefore quantified the response of the selected cell populations after PDT, to take into account tumor heterogeneity. Since the total number of stem‐like cells following selection is in the 100–1000 range, we used the sensitive alamar blue cell viability assay rather than the MTT assay 26. We showed the sensitivity of alamar blue to be suitable for 500 cells per well (Fig. S2). This assay was also multiplexed with the ROS‐Glo assay to measure H_2_O_2_. Following PDT, there was an increase in ROS production in all cell types (Fig. 3A), which correlated with a decrease in cell viability (Fig. 3B).

### Differential oxidative stress gene expression is induced in cancer versus benign cells

When comparing the different disease states in ROS and viability assays there was a noticeable difference between cancer and BPH samples (Fig. S3). The cancer samples appeared to have both increased reactive oxygen species but also increased viability. The difference was observed in all cases but was only statistically significant in CB cells in the ROS‐Glo assay and in TA cells in the cell viability assay. In order to investigate this observation further, we carried out a reanalysis of our published microarray data 27 that compared gene expression in stem‐like cells (SC) and committed basal cells (CB) in both BPH and cancer patient samples. We analyzed 84 genes that are associated with response to oxidative stress (list obtained from the QIAGEN Oxidative Stress RT 2 Profiler PCR Arrays). We found that there was significant differential gene expression between BPH and Cancer (Table 2). Several of the genes that were overexpressed in benign samples compared to cancer encode for antioxidants, and play a role in protecting cells from oxidative damage, including conversion of H_2_O_2_ to water and oxygen (e.g., genes encoding for glutathione peroxidases, catalase and a peroxiredoxin, and ATOX1, and SCARA3). In addition, MSRA (involved in repairing oxidative damage to proteins) and NQO1 (prevents production of radical species) were also differentially expressed. Finally, EPAS1, a transcription factor that activates genes, including hypoxia responsive genes following reduction in oxygen levels.

**Table 2:** Oxidative stress genes are differentially expressed in cancer and benign cells

Significance	Higher in benign	Higher in cancer
<0.01	GPX3	APOE
	GPX4	ALOX12
	GPX7	NOX4
	CAT	SFTPD
	ATOX1	
<0.05	PRDX6	APOE^a^
	TTN	NOX5
	GCLC	BNIP3
	MSRA	
	NQO1	
	SCARA3	
<0.1	GPX2	PXDN
	GCLC^a^	NCF2
	SQSTM1	NOX4^a^
	EPAS1	UCP2
		BNIP3^a^
		FTH1
		FOXO4

a Genes that have more than one probe.

Conversely to the genes overexpressed in BPH, several genes that were overexpressed in cancer compared to benign cells produce ROS, rather than quenching these species. For example, NOX4 senses oxygen, produces ROS and is associated with tumor growth, and NOX5 and NCF2 produce superoxide. APOE and ALOX12 are involved in lipid metabolism, whereas SFTPD is involved in the innate immune response to interacting with sugars and fats of pathogens. BNIP3 has both antiapoptotic and proapoptotic functions. UCP2 is a mitochondrial transporter protein and is involved in control of reactive oxygen species derived in the mitochondria, FTH1 encodes for the heavy subunit of ferritin, and FOXO4 suppresses expression of hypoxia‐induced genes.

### PDT drug induces DNA damage, autophagy, and necrosis, whereas gamma irradiation induces senescence

**Figure 4:**
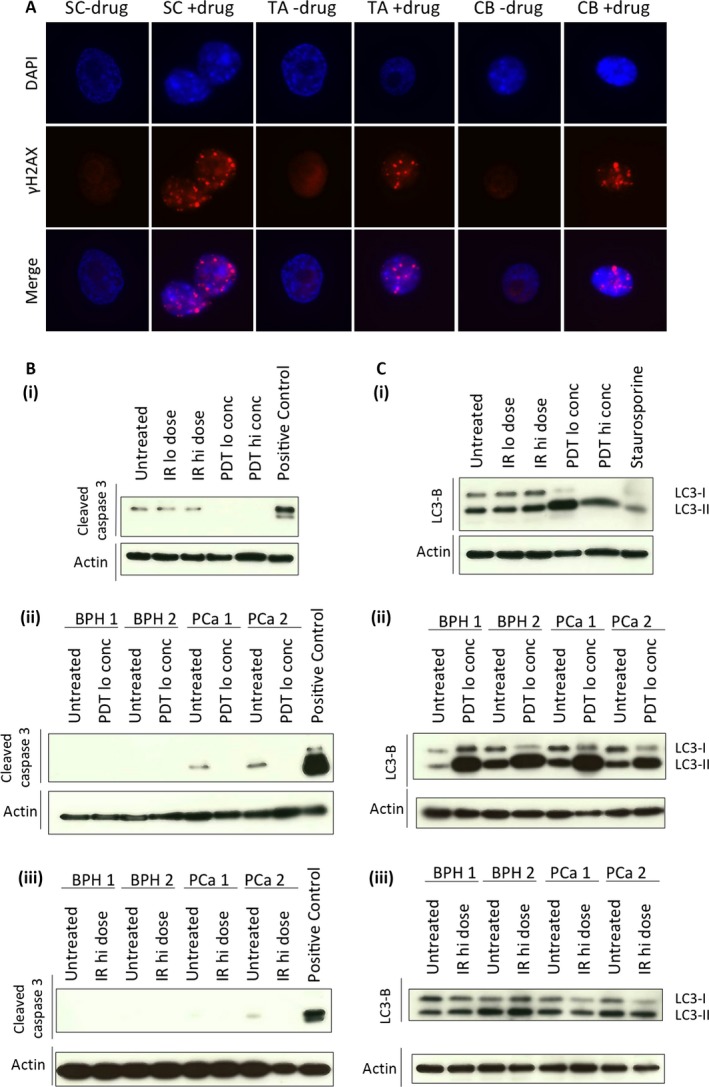
Neither photodynamic therapy (PDT) drug nor gamma irradiation (IR) induces apoptosis, whereas PDT drug induces DNA damage and autophagy. (A) Selected primary prostate epithelial cell populations were treated with PDT drug (12.5 *μ*mol/L) and assessed for DNA damage, observed as gamma‐H2AX foci. Primary prostate epithelial cells from four patients (2× Benign prostatic hyperplasia (BPH) and 2 × PCa) were treated with PDT drug or gamma irradiation and a western blot for cleaved caspase 3 was carried out to detect (B) apoptosis or (C) LC3‐B to detect autophagy. (PDT drug lo = 12.5 *μ*mol/L per hi = 50 *μ*mol/L; Gamma irradiation lo = 2 Gy/hi = 50 Gy).

We had previously observed induction of cytotoxic DNA damage in the majority of primary prostate epithelial cells following gamma irradiation treatment. After PDT‐treatment of cells, we were also able to demonstrate DNA damage in all cell types (SC, TA, CB) (Fig. 4A).

Since the two therapies showed different results in the cell viability assays, but both showed reduction in colony‐forming ability, we then examined the consequences of the treatments on the cells. Firstly, we measured apoptosis in one cancer sample and showed that neither PDT (low and high conc) nor gamma irradiation (low and high dose) resulted in cleaved caspase 3 formation (Fig. 4B(i)). This negative result was confirmed in three further patient samples (two BPH and two cancer total), where apoptosis was not induced by PDT (Fig. 4B(ii)) or gamma irradiation (Fig. 4B(iii)) in any sample. We next investigated a potential autophagic response following treatment. Here, PDT effectively induced autophagy in one cancer sample (Fig. 4C(i)), indicated by reduction in levels of LC3‐I and increase in levels of LC3‐II. In three further samples (two BPH and two cancer total) following PDT drug treatment (Fig. 4C(ii)) or gamma irradiation (Fig. 4C(iii)) treatment, the PDT drug, but not gamma irradiation, induced autophagy.

**Figure 5:**
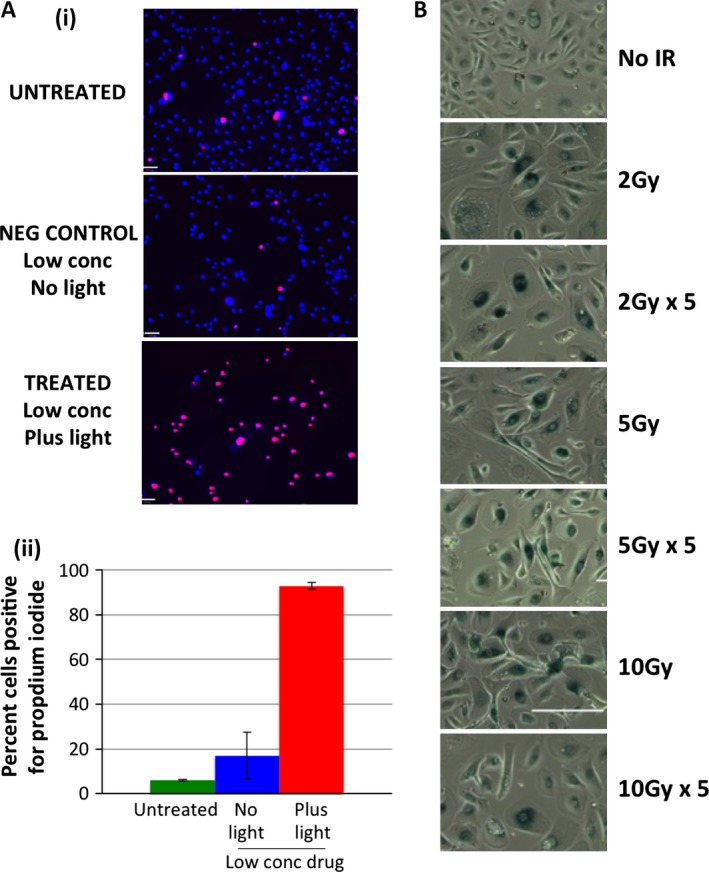
Gamma irradiation induces senescence in primary prostate epithelial cells, whereas photodynamic therapy (PDT) drug induces necrosis. (A) Primary prostate epithelial cells were treated with PDT drug (12.5 *μ*mol/L) and (i) stained for necrotic cells (Propidium iodide positive) then (ii) percentage necrotic cells was measured by expressing Propidium iodide positive cells relative to total cells (DAPI positive). (B) Primary prostate epithelial cells were treated with single or multiple doses of gamma irradiation as indicated, incubated for 48 h and stained for beta‐galactosidase.

The PDT‐treated cultures revealed considerable cytotoxicity (floating or damaged cells), and were assessed for necrosis (Fig. 5A). This was found to be the predominant PDT‐induced death mechanism in primary prostate epithelial cells. In contrast, the gamma‐irradiation‐treated cells appeared intact, and neither damaged nor dead. However, the cells had flattened and morphologically appeared either terminally differentiated or senescent. After treatment with single and multiple doses of gamma irradiation, senescence in primary prostate epithelial cells was apparent (Fig. 5B). This provides an explanation for both the minimal reduction in cell survival in the cell viability assay (Fig. 1C) and the lack of colony‐forming ability (Fig. 2B).

## Discussion

Since the established standard of care for prostate cancer (gamma irradiation) and the more recent development of PDT both generate toxic levels of ROS, it may have been assumed that they would achieve their therapeutic killing in patient cells by the same mechanism. However, this study presents evidence to show that PDT and gamma irradiation in fact inhibit cell growth by very different mechanisms. Our data indicate that irradiation of a photosensitizing agent with light irradiation (PDT) inhibits growth of prostate cells cultured from patient samples, which are a clinically relevant model 28. Since the initial cell viability assays showed a significant decrease in the primary cells, we also determined long‐term recovery of cells following treatment, using colony‐forming assays. The data indicated a dose‐dependent reduction in colony formation, where no colonies formed after treatment with 50 *μ*mol/L of PDT drug in all six primary cell cultures. At 37.5 *μ*mol/L and 25 *μ*mol/L there were also no or minimal colonies seen in all patient cultures. Interestingly, while gamma irradiation (2–75 Gy) did not reduce cell viability by more than 20%, there was a significant effect of gamma irradiation on colony‐forming ability. A potent inhibition in colony‐forming ability was seen after doses of 10 Gy and above.

In order to take into account cell heterogeneity within the primary prostate epithelial cell cultures, representative of tumor heterogeneity in the patients, we sorted cells and treated each cell subpopulation with the PDT drug, determining both viability and the production of ROS. In other cancers, ROS‐quenching enzymes have been implicated as a therapy resistance mechanism in cancer stem cells 29, although there is no evidence for this in prostate cancer. After first establishing that there was a dose‐dependent production of H_2_O_2_ following addition of the PDT drug, the sorted cell populations were tested. We were able to multiplex the ROS‐Glo assay with the alamar blue assay to maximize data from a very small number (100s) of cells. In primary prostate epithelial cells we showed a correlation between the increase in ROS, and a loss of viability in all three cell populations (SC, TA, CB).

After interrogating gene expression microarray data comparing stem‐like cells and committed basal cells from benign and malignant patient tissue 27, we found that benign cells expressed genes that are involved in quenching ROS, whereas cancer cells expressed genes encoding products that result in production of ROS. It is known that cancer cells typically have increased levels of ROS, which can act to boost mutation frequency 30, 31, 32. This is the logic behind using ROS producing agents as therapies, to increase the amount of ROS production in cancer cells over a tolerance‐threshold, resulting in increased cell death 33. ROS production in cancer cells is dichotomous, since it can be both an inducer of cell death, but can also promote cancer cell proliferation 34, 35. The increase in genes related to ROS quenching in benign cells suggests that these mechanisms are intact in benign cells, whereas they will either be absent in cancer cells or overridden by the ROS‐producing mechanisms.

Since the photosensitizer induced ROS in all cell subpopulations from the primary prostate epithelial cultures, leading to a reduction in cell viability and colony‐forming ability, (attributable to induction of necrosis Fig. 5A), this drug seems ideally suited for use in prostate cancer treatment. Although both gamma irradiation and PDT induce DNA damage, the consequences for the cell are different in each case. The induction of autophagy we observed (Fig. 4C) is interesting because it can act as a cell‐protective mechanism 36, but can also lead to cell death 37.

Since the drug in this study can be conjugated to peptides and antibodies, future studies using these compounds will investigate improved photosensitizer targeting of prostate cancer cells. Increased targeting of the photosensitizer for use in prostate cancer patients can now be attempted by binding the drug to a prostate‐specific antibody 38. Alternatively, nanoparticle technology could be explored to deliver the drug more precisely 39. Another approach is to target the antibody and the drug to the vasculature and once light irradiation is directed to the tumor this will selectively destroy the tumor vasculature and result in cell and tumor death. Promising results using this method have already been demonstrated in xenograft models of teratocarcinoma and epidermoid carcinoma cells 20. Finally, targeting established hypoxia‐associated surface markers may be a strategy to impact the cells in the center of the tumor.

As a comparison with the consequences of the photosensitizer treatment, we carried out gamma irradiation treatments, which we have previously shown to reduce the colony‐forming ability of primary prostate epithelial cells 22. Interestingly, this was the caused by senescence induction (Fig. 5B) rather than apoptosis, necrosis or autophagy 40, 41. Lack of induction of these cell death mechanisms may partially explain why there is frequent radio‐recurrence of prostate cancer 23.

The absence of apoptosis is potentially unexpected, since PDT is known to induce apoptosis in some cell lines 42, 43, 44. However, previous studies on the effects of chemotherapeutic drugs on primary prostate cells compared to established cell lines, showed that apoptosis was not induced in the primary cells 28. In addition, after treatment of primary cell cultures with another cytotoxic agent, low‐temperature plasma, which also creates reactive species, cell death was also induced by necrosis and not apoptosis 45.

The basis of the profound differences between PDT and gamma irradiation treatments may lie in the mechanisms of ROS generation. Gamma irradiation causes DNA damage either directly through chemical modification of the DNA molecule or indirectly via the production of reactive oxygen species 46. However, PDT causes DNA damage predominantly in an indirect manner through production of ROS and cytotoxic intermediate molecules in the cytoplasm following light activation 47.

Here, we present evidence in near‐patient models that both gamma irradiation and PDT induce production of reactive oxygen species, but result in differential cytotoxic responses. The data imply that a combination of strategies would provide a complementary spectrum of cytotoxicity. Some studies have already used them in combination 48 as part of salvage therapy or pretreatment 15, 49, 50. Due to technical difficulties in accurately delivering both radiation and light to patients simultaneously, it is most likely that the treatments would be administered sequentially, as shown in other tumor types 51. By providing evidence for mechanisms of cell growth inhibition or cell death, there are now sound biological reasons to promote the use of such combination therapies in the clinic.

## Conflict of Interest

None declared.

## Supporting information


**Figure S1.** Increasing doses of PDT drug or gamma irradiation results in a reduction and ablation of colony‐forming ability of primary prostate epithelial cells.

**Figure S2.** Alamar blue is a sensitive cell viability assay.

**Figure S3.** Treatment of selected populations of primary prostate epithelial cells with one dose of PDT drug shows differential results between benign and cancer samples.
