# Supplementary material for: Mechanisms of growth inhibition of primary prostate epithelial cells following gamma irradiation or photodynamic therapy include senescence, necrosis, and autophagy, but not apoptosis
Source: Cancer Med. 2015 Nov 21;5(1):61–73. doi: 10.1002/cam4.553 (PMC4708897; doi:10.1002/cam4.553)
Supplement: Supplementary file 1 — Figure S1. Increasing doses of PDT drug or gamma irradiation results in a reduction and ablation of colony‐forming ability of primary prostate epithelial cells. [file CAM4-5-061-s001.pptx]

## Slide 1
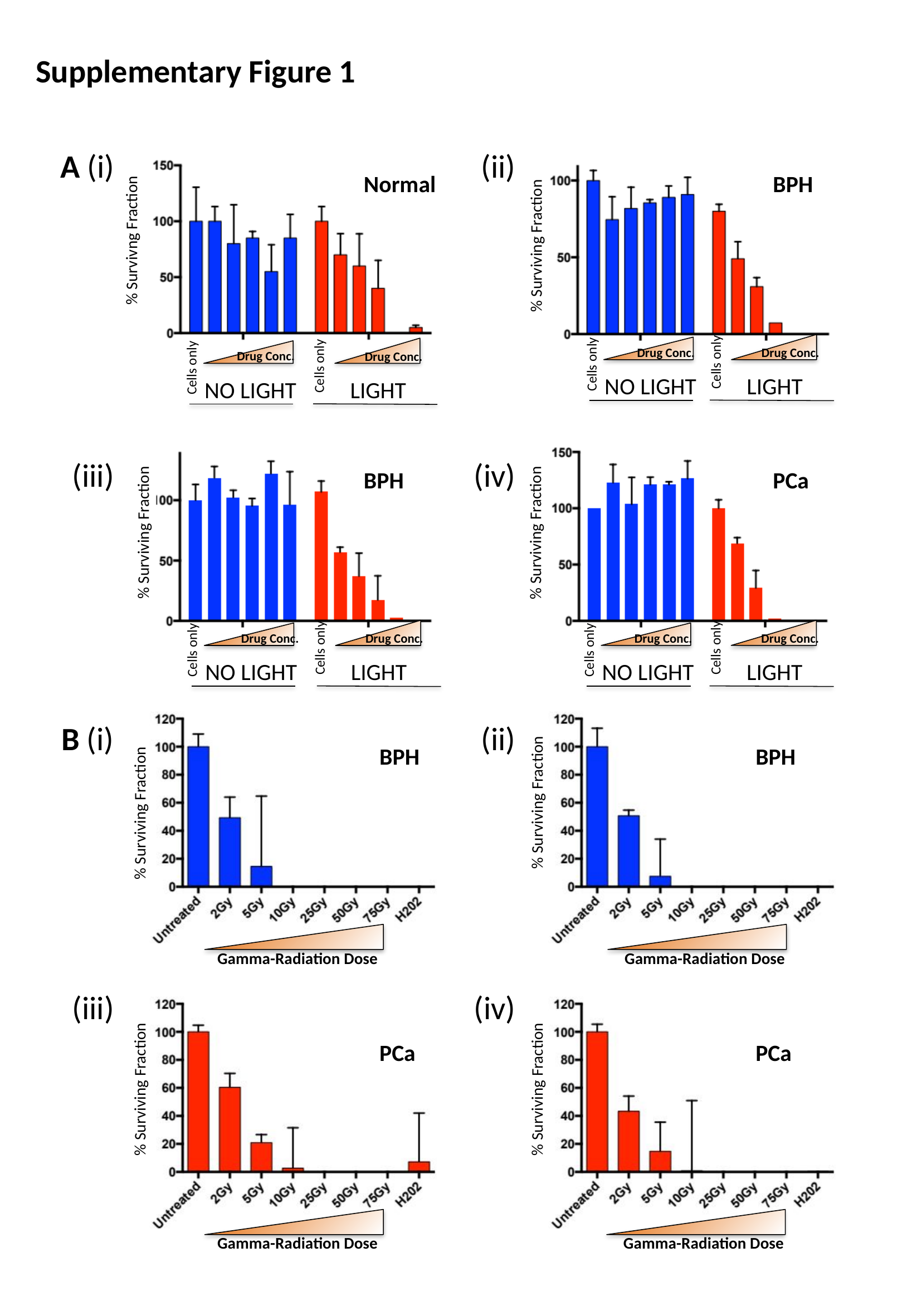

Supplementary Figure 1
A (i)
 (ii)
Normal
BPH
% Survivng Fraction
% Surviving Fraction
Drug Conc.
Drug Conc.
Drug Conc.
Drug Conc.
Cells only
Cells only
Cells only
Cells only
NO LIGHT
LIGHT
NO LIGHT
LIGHT
(iii)
(iv)
BPH
PCa
% Surviving Fraction
% Surviving Fraction
Drug Conc.
Drug Conc.
Drug Conc.
Drug Conc.
Cells only
Cells only
Cells only
Cells only
NO LIGHT
LIGHT
NO LIGHT
LIGHT
B (i)
 (ii)
BPH
BPH
% Surviving Fraction
% Surviving Fraction
Gamma-Radiation Dose
Gamma-Radiation Dose
(iii)
(iv)
PCa
PCa
% Surviving Fraction
% Surviving Fraction
Gamma-Radiation Dose
Gamma-Radiation Dose
