# Supplementary material for: Mechanisms of growth inhibition of primary prostate epithelial cells following gamma irradiation or photodynamic therapy include senescence, necrosis, and autophagy, but not apoptosis
Source: Cancer Med. 2015 Nov 21;5(1):61–73. doi: 10.1002/cam4.553 (PMC4708897; doi:10.1002/cam4.553)
Supplement: Supplementary file 2 — Figure S2. Alamar blue is a sensitive cell viability assay. [file CAM4-5-061-s002.pptx]

## Slide 1
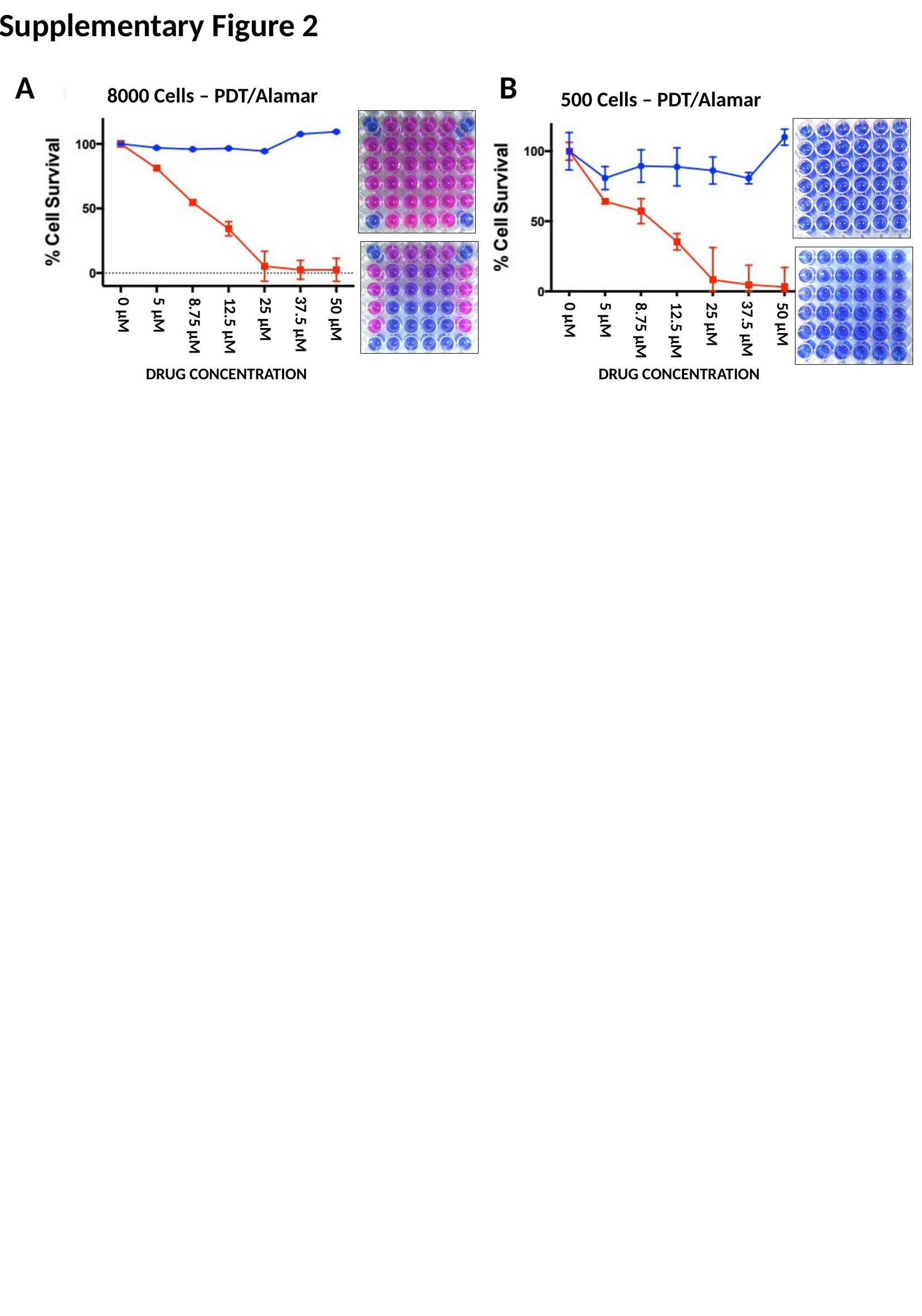

Supplementary Figure 2
A
B
 8000 Cells – PDT/Alamar
 500 Cells – PDT/Alamar
0 μM
5 μM
25 μM
50 μM
0 μM
5 μM
25 μM
50 μM
8.75 μM
12.5 μM
37.5 μM
8.75 μM
12.5 μM
37.5 μM
DRUG CONCENTRATION
DRUG CONCENTRATION
