# Supplementary material for: Mechanisms of growth inhibition of primary prostate epithelial cells following gamma irradiation or photodynamic therapy include senescence, necrosis, and autophagy, but not apoptosis
Source: Cancer Med. 2015 Nov 21;5(1):61–73. doi: 10.1002/cam4.553 (PMC4708897; doi:10.1002/cam4.553)
Supplement: Supplementary file 3 — Figure S3. Treatment of selected populations of primary prostate epithelial cells with one dose of PDT drug shows differential results between benign and cancer samples. [file CAM4-5-061-s003.pptx]

## Slide 1
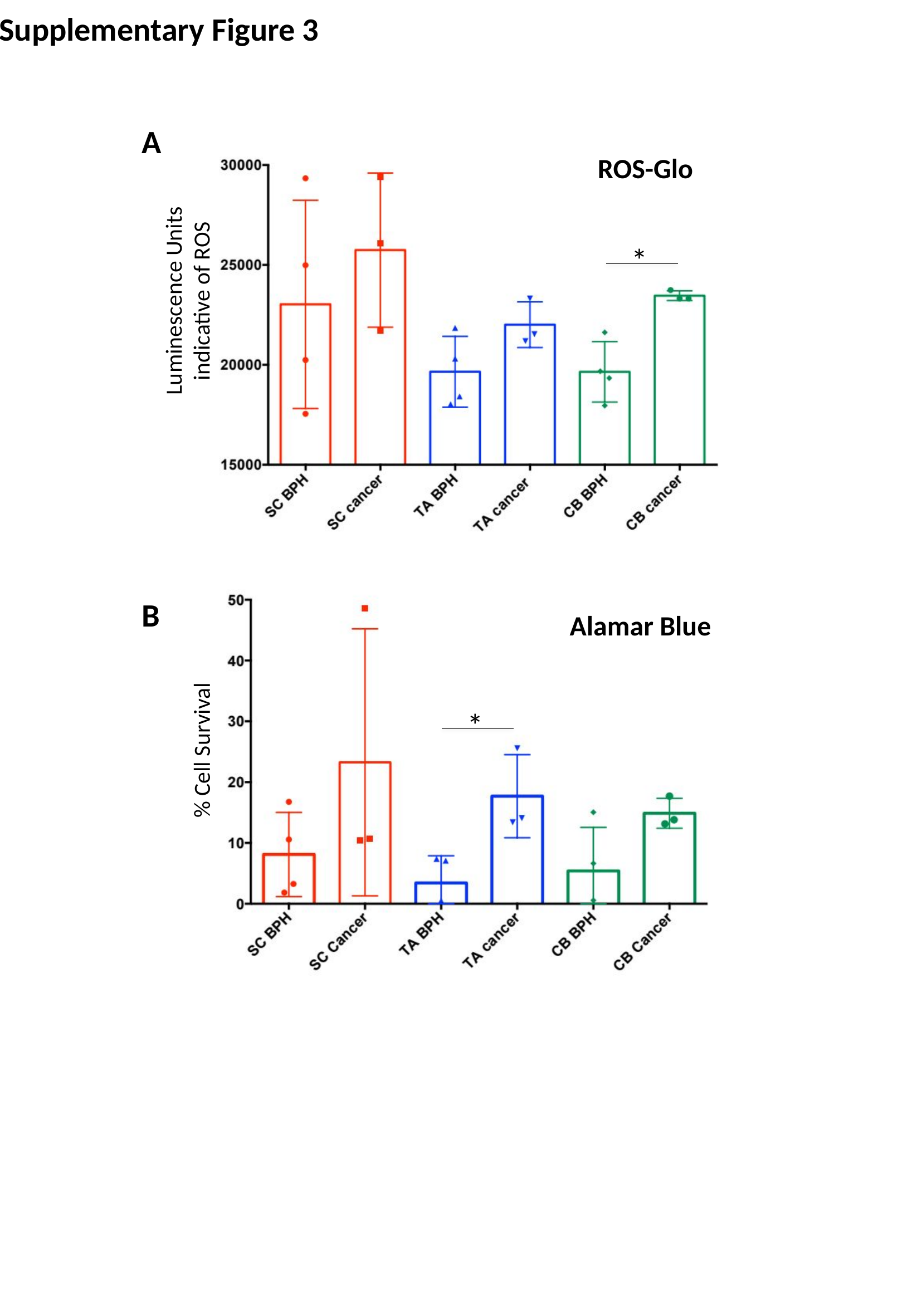

Supplementary Figure 3
A
ROS-Glo
*
Luminescence Units
indicative of ROS
B
Alamar Blue
*
% Cell Survival
